# Epidemiological, Clinical and Laboratory Characteristics of Hospitalized Children with SARS-CoV-2 Infection During Three Consecutive Pandemic Years: A Retrospective Cohort Study

**DOI:** 10.3390/children13101423

**Published:** 2026-10-16

**Authors:** Ioana Maria Otilia Lică, Iulia Florentina Țincu, Anca Cristina Drăgănescu, Alexandru Dinulescu, Doina Anca Pleșca

**Affiliations:** 1 Pediatric Department, University of Medicine and Pharmacy “Carol Davila”, 050474 Bucharest, Romania; ioana-maria-otilia.lica@drd.umfcd.ro (I.M.O.L.); alexandru.dinulescu@drd.umfcd.ro (A.D.);; 2 “Dr. Victor Gomoiu” Children’s Clinical Hospital, 022102 Bucharest, Romania; 3 National Institute of Infectious Diseases “Prof. Dr. Matei Balș”, 021105 Bucharest, Romania; 4 Emergency Hospital for Children ‘’Grigore Alexandrescu”, 011743 Bucharest, Romania

**Keywords:** SARS-CoV-2, pediatrics, epidemiology, clinical characteristics, disease severity, laboratory characteristics

## Abstract

**Highlights:**

**What are the main findings?**

- Pediatric COVID-19 showed a distinct temporal evolution across the first three pandemic years, with significant changes in epidemiological characteristics, clinical presentation, and laboratory profiles among hospitalized children.
- Respiratory involvement became increasingly prominent, rising from 61.9% in 2020 to 77.3% in 2022, while gastrointestinal manifestations remained frequent and cardiovascular manifestations were reported in 28.9%, highlighting the evolving multisystem clinical phenotype of pediatric SARS-CoV-2 infection.

**What is the implication of the main finding?**

- Pediatric COVID-19 should not be regarded as a static clinical entity, as its phenotype changed substantially across successive stages of the pandemic.
- Recognizing these temporal changes may improve the interpretation of pediatric SARS-CoV-2 presentations and support continued age- and period-specific clinical surveillance of hospitalized children.

**Abstract:**

Background: Coronavirus disease 2019 (COVID-19) generally follows a milder clinical course in children than in adults. The clinical characteristics of pediatric severe acute respiratory syndrome coronavirus 2 (SARS-CoV-2) infection have evolved throughout the pandemic and longitudinal data comparing successive pandemic years remain limited. Objective: To compare the epidemiological, clinical, and laboratory characteristics of children hospitalized with SARS-CoV-2 infection during 2020–2022 and to identify factors associated with disease severity. Methods: We conducted a retrospective cohort study of children aged 1 month to 18 years hospitalized with laboratory-confirmed SARS-CoV-2 infection at a tertiary pediatric hospital in Bucharest, Romania, between January 2020 and December 2022. Results: Of 669 eligible patients, 519 met the inclusion criteria (118 in 2020, 168 in 2021, and 233 in 2022). The proportion of hospitalized infants increased over time, whereas adolescent admissions declined (*p* < 0.001). Respiratory manifestations remained predominant, while gastrointestinal involvement was consistently common. Respiratory manifestations increased from 61.9% in 2020 to 77.3% in 2022 (*p* = 0.01), while gastrointestinal manifestations remained consistently frequent. Cardiovascular manifestations were reported in 28.9% of patients, without significant differences across the study years (*p* = 0.06). Neuromuscular manifestations declined from 43.2% in 2020 to 12.0% in 2022 (*p* < 0.001). Leukocyte count, lymphocyte count, and hemoglobin differed significantly across the study period, whereas C-reactive protein remained unchanged. Moderate-to-severe disease was associated with digestive, cardiovascular, and reno-urinary involvement but not with sex or residence. Conclusions: The epidemiological and clinical profile of hospitalized children with SARS-CoV-2 infection changed substantially across the first three years of the pandemic. These findings highlight the importance of continued surveillance and comprehensive clinical assessment to optimize the management of pediatric COVID-19.

## 1. Introduction

Since its emergence in late 2019, severe acute respiratory syndrome coronavirus 2 (SARS-CoV-2), the causative agent of coronavirus disease 2019 (COVID-19), has had a profound impact on pediatric healthcare worldwide. Although children generally experience a milder clinical course than adults, SARS-CoV-2 infection in the pediatric population is characterized by considerable clinical heterogeneity [1,2,3,4,5]. SARS-CoV-2 is primarily transmitted through inhalation of infectious respiratory particles, including droplets and aerosols released during breathing, speaking, coughing, or sneezing [6]. Transmission through direct contact with infected individuals or indirect contact with contaminated surfaces has also been described, but these routes are considered less important [7]. The detection of viral ribonucleic acid (RNA) in stool samples and evidence of gastrointestinal infection have also raised the possibility of fecal–oral transmission, but the epidemiological significance of this potential route remains uncertain. Although not yet widely recognized, the fecal-oral route should be taken into consideration [8,9].

Most children develop asymptomatic or mild disease, whereas others require hospitalization because of pneumonia, respiratory failure, multisystem inflammatory syndrome in children (MIS-C), or other severe complications. While fatal outcomes remain uncommon, they have been reported, particularly among children with underlying medical conditions [10,11,12,13,14,15]. As evidence accumulated throughout the pandemic, it became increasingly clear that pediatric COVID-19 cannot be regarded as a uniformly benign disease. Younger age, pre-existing comorbidities, and host-related factors have consistently been associated with an increased risk of hospitalization and severe clinical outcomes, highlighting the importance of continuously characterizing the evolving spectrum of SARS-CoV-2 infection in children [14,15,16]. Although respiratory symptoms remain the predominant manifestation of pediatric COVID-19, it is now well established that SARS-CoV-2 infection may affect multiple organ systems [17]. Hospitalized children may present with gastrointestinal, neurological, cardiovascular, renal, or dermatological involvement, either in association with respiratory disease or, less frequently, as the predominant clinical presentation [17,18,19,20,21]. This clinical heterogeneity reflects the complex pathophysiology of SARS-CoV-2 infection and contributes to the wide variation in disease severity observed among pediatric patients [17,18].

The clinical profile of pediatric COVID-19 has evolved considerably throughout the pandemic. The emergence of successive SARS-CoV-2 variants, together with increasing population immunity resulting from previous infections and vaccination, altered patterns of viral transmission, age distribution, and disease presentation over time [22,23,24,25,26]. Although later variants were generally associated with milder clinical manifestations, their markedly increased transmissibility led to a substantial rise in the absolute number of infected and hospitalized children [19,27,28,29]. Several studies, including those conducted in Romania, have investigated the clinical characteristics and severity of pediatric COVID-19 during different stages of the pandemic, providing valuable insights into changes in disease presentation over time [30,31,32]. Additional data from different pediatric cohorts and healthcare settings may contribute to a more comprehensive understanding of these changes.

Therefore, this study aimed to compare the epidemiological characteristics, clinical manifestations, laboratory findings, and disease severity of children hospitalized with laboratory-confirmed SARS-CoV-2 infection during three consecutive pandemic years (2020–2022) in a tertiary pediatric referral center. By evaluating temporal changes in disease presentation within a single-center cohort, we sought to characterize the evolving clinical phenotype of pediatric COVID-19 and identify factors associated with disease severity.

## 2. Materials and Methods

### 2.1. Study Design and Setting

This retrospective observational cohort study included children aged 1 month to 18 years hospitalized with laboratory-confirmed acute SARS-CoV-2 infection at the “Dr. Victor Gomoiu” Children’s Clinical Hospital, a tertiary pediatric referral center in Bucharest, Romania, between January 2020 and December 2022. The study aimed to compare the epidemiological characteristics, clinical manifestations, laboratory findings, disease severity, and outcomes of pediatric SARS-CoV-2 infection across the first three years of the COVID-19 pandemic. Clinical, epidemiological, laboratory, imaging, therapeutic, and outcome data were extracted from hospital medical records using a standardized data collection form.

### 2.2. Inclusion and Exclusion Criteria

Children aged 0–18 years hospitalized at “Dr. Victor Gomoiu” Children’s Clinical Hospital between 2020 and 2022 with laboratory-confirmed SARS-CoV-2 infection were eligible for inclusion in this retrospective study. Patients with incomplete medical records that did not allow adequate extraction of the variables required for analysis were excluded. Following the application of these criteria, a total of 519 patients were included in the final study cohort.

### 2.3. Variables

The following variables were analyzed: demographic characteristics (age, sex, year of hospitalization, and area of residence), underlying comorbidities, clinical manifestations, laboratory parameters, imaging findings, and clinical outcomes, including duration of hospitalization, oxygen supplementation, pediatric intensive care unit (PICU) admission, complications, and discharge status. Laboratory investigations included complete blood count, inflammatory markers, liver enzymes, coagulation parameters, and other tests performed according to clinical indication. Imaging investigations were recorded when clinically indicated.

### 2.4. Statistical Analysis

Data were analyzed using IBM SPSS Statistics version 30.0 (IBM Corp., Armonk, NY, USA). Categorical variables were expressed as absolute frequencies and percentages. Comparisons of categorical variables across the three study years (2020, 2021, and 2022), as well as between male and female patients, were performed using Pearson’s chi-square test. Standard errors (SE) were calculated for proportions using the binomial formula to provide an estimate of their precision.

Cases with missing values for a specific variable were excluded using complete-case (listwise) analysis. Missing data accounted for less than 5% of the dataset; therefore, no imputation procedures were performed.

All statistical tests were two-sided, and a *p*-value < 0.05 was considered statistically significant.

### 2.5. Ethical Approval

The retrospective study was approved by the Institutional Ethics Committee of the “Dr. Victor Gomoiu” Children’s Clinical Hospital, Bucharest, Romania (approval no. 6648/3 April 2024). Written informed consent for the use of anonymized medical data for research purposes was obtained from parents or legal guardians at the time of hospital admission. All patient data were anonymized before statistical analysis in accordance with the Declaration of Helsinki and applicable data protection regulations.

## 3. Results

A total of 669 pediatric patients with laboratory-confirmed SARS-CoV-2 infection were initially identified at the “Dr. Victor Gomoiu” Children’s Clinical Hospital between January 2020 and December 2022. After applying the predefined inclusion and exclusion criteria, 519 children were included in the final analysis.

The study cohort comprised 118 patients (22.7%) hospitalized in 2020, 168 (32.4%) in 2021, and 233 (44.9%) in 2022.

### 3.1. Demographic and Epidemiological Characteristics

Age distribution differed significantly across the three study years (*p* < 0.001). The proportion of infants aged <1 year increased progressively from 33.9% in 2020 to 48.2% in 2021, reaching 60.9% in 2022. In contrast, the proportion of adolescents aged 10–18 years declined from 20.3% in 2020 to 13.7% in 2021 and 4.7% in 2022 (Table 1).

The sex distribution remained stable throughout the study period, with male patients accounting for 61.9%, 57.1%, and 57.5% of hospitalized children in 2020, 2021, and 2022, respectively, without statistically significant differences between years (*p* = 0.681).

Children from urban areas represented the majority of hospitalized patients throughout the study period. The proportion of urban residents was highest in 2022 (71.7%), compared with 66.9% in 2020 and 58.3% in 2021, with a statistically significant difference between study years (*p* = 0.020).

Hospitalization duration also varied significantly across the three years (*p* < 0.001). In 2020, most patients were hospitalized for 1–3 days (89.0%), whereas hospitalizations exceeding seven days were most frequent in 2021 (40.5%). In 2022, stays lasting 4–7 days predominated, accounting for 59.7% of admissions.

The proportion of patients reporting household contact with a confirmed SARS-CoV-2 case was 50.8% in 2020, 50.6% in 2021, and 48.5% in 2022, with no statistically significant differences in the distribution of epidemiological contact categories across the three study years (*p* = 0.14) (Table 1).

The sex-stratified analysis revealed no statistically significant differences in the distribution of patients across study years (*p* = 0.681), age groups (*p* = 0.503), or residence (*p* = 0.414). Infants younger than one year represented the largest age group in both sexes, accounting for 50.0% of female patients and 51.2% of male patients.

Hospitalization duration was also comparable between female and male patients (*p* = 0.170). Stays lasting 1–3 days were recorded in 36.1% of female patients and 43.6% of male patients, while hospitalizations lasting 4–7 days occurred in 44.9% and 37.3%, respectively. Prolonged hospitalizations exceeding seven days were observed in similar proportions of female and male patients (19.0% versus 19.1%). Overall, no statistically significant sex-related differences were identified in the demographic or hospitalization characteristics evaluated (Table 2).

### 3.2. Clinical Characteristics

Clinical characteristics differed significantly across the three study years. The prevalence of fever increased progressively over time, reaching its highest frequency in 2022, from 75.4% in 2020 to 81.0% in 2021 and 89.3% in 2022 (*p* = 0.003). Fever duration also differed significantly between years (*p* = 0.003), with the shortest duration observed among children hospitalized in 2020. Fever lasting 1–3 days was reported in 65.3% of patients in 2020, 60.7% in 2021, and 75.1% in 2022, while fever lasting more than three days was observed in 10.2%, 20.2%, and 12.9% of patients, respectively.

The overall clinical condition at admission varied significantly throughout the study period (*p* < 0.001). The proportion of patients presenting with a moderate clinical condition increased from 3.4% in 2020 to 39.9% in 2021 and 42.9% in 2022, while the proportion of patients with a satisfactory clinical condition decreased from 89.0% to 58.9% and 55.4%, respectively.

Similarly, peripheral oxygen saturation (SpO_2_) differed significantly between study years (*p* < 0.001). The proportion of patients with SpO_2_ > 95% increased from 78.0% in 2020 to 86.3% in 2021 and 94.4% in 2022, while the proportion of patients with SpO_2_ between 90% and 94% decreased from 20.3% to 13.1% and 5.6%, respectively. The distribution of clinical manifestations across organ systems varied during the study period (Table 3). Respiratory manifestations increased significantly from 61.9% in 2020 to 70.2% in 2021 and 77.3% in 2022 (*p* = 0.01). Conversely, neuromuscular manifestations decreased significantly, from 43.2% in 2020 to 21.4% in 2021 and 12.0% in 2022 (*p* < 0.001). Gastrointestinal manifestations remained frequent throughout the study period, with comparable proportions in 2020 (75.4%), 2021 (74.4%), and 2022 (78.1%; *p* = 0.66). Cardiovascular manifestations were reported in 37.3%, 25.0%, and 27.5% of patients, respectively, without a statistically significant difference (*p* = 0.06). Similarly, no statistically significant differences were observed for reno-urinary manifestations (*p* = 0.12) or cutaneous eruptions (*p* = 0.12).

Detailed comparative clinical characteristics are presented in Table 3.

### 3.3. Laboratory Findings

Paraclinical findings demonstrated significant temporal changes across the three study years. Significant differences were observed in hematological parameters. Total leukocyte counts were highest in 2020 (median 10,988.5 cells/µL), with significantly lower values recorded in both 2021 (7395 cells/µL) and 2022 (7000 cells/µL) (*p* < 0.001). In contrast, lymphocyte counts progressively decreased during the study period, reaching their lowest values in 2022 (median 2770 cells/µL; *p* = 0.03). Hemoglobin concentrations also differed significantly across the three years, decreasing from 11.8 g/dL in 2020 to 11.2 g/dL in 2021 and 10.9 g/dL in 2022 (*p* = 0.02), with the lowest median values observed in 2022, indicating a higher frequency of anemia among hospitalized children.

No statistically significant differences were identified in absolute neutrophil counts (*p* = 0.45) or C-reactive protein (CRP) concentrations (*p* = 0.08), suggesting that the overall inflammatory response remained comparable throughout the study period despite changes in patient demographics and clinical presentation.

The comparative laboratory characteristics of pediatric patients hospitalized with SARS-CoV-2 infection during the three study years are summarized in Table 4.

### 3.4. Analysis of Comorbidities

The prevalence of underlying comorbidities differed significantly across the three study years (*p* < 0.001; Table 5). The proportion of children without pre-existing medical conditions was 66.9% in 2020, 61.3% in 2021, and 82.8% in 2022, accounting for 72.3% of the overall study population.

Neurological disorders represented the most common category of comorbidities, affecting 7.5% of patients overall, followed by chronic gastrointestinal diseases (6.4%), cardiovascular disorders (4.0%), respiratory diseases (3.3%), and immunological or hematological (3.3%). Less frequently encountered conditions included psychiatric disorders (1.7%), reno-urinary diseases (1.3%), diabetes or obesity (1.0%), ophthalmological disorders (0.6%), and genetic syndromes (0.4%).

The prevalence of gastrointestinal comorbidities differed significantly across the study years, being reported in 7.6% of patients in 2020, 9.5% in 2021, and 3.4% in 2022 (*p* = 0.039). Neurological disorders decreased in frequency from 11.9% in 2020 to 7.1% in 2021 and 5.6% in 2022, although this difference was not statistically significant (*p* = 0.105). Similarly, immunological, hematological, or genetic disorders were reported in 2.5%, 6.0%, and 1.7% of patients, respectively, without reaching statistical significance (*p* = 0.055).

Ophthalmological disorders were uncommon, being identified exclusively in 2021 (1.8%; *p* = 0.043). No statistically significant differences were observed for the remaining comorbidity categories across the study period.

## 4. Discussion

This longitudinal cohort study provides a comprehensive overview of the evolving epidemiological, clinical, and laboratory characteristics in children hospitalized with SARS-CoV-2 infection during the first three years of the COVID-19 pandemic. Although the overall clinical course remained predominantly mild to moderate, we observed significant temporal changes in patient demographics, clinical presentation, laboratory parameters, and the prevalence of underlying comorbidities. These findings indicate that the clinical phenotype of pediatric COVID-19 was not static but evolved throughout successive pandemic waves. Similar temporal trends have been described in large multinational pediatric cohorts, where changes in circulating SARS-CoV-2 variants, increasing population immunity, vaccination, and evolving healthcare policies influenced both disease presentation and hospitalization patterns [24,25,33,34]. Together, these observations suggest that the differences identified in our cohort most likely reflect the combined effects of viral evolution and changing epidemiological conditions rather than substantial differences in the intrinsic severity of pediatric SARS-CoV-2 infection.

We observed a marked shift in the demographic profile of hospitalized children during the study period, with infants becoming the predominant age group by 2022 and adolescent admissions progressively declining. The proportion of infants aged <1 year increased from 33.9% in 2020 to 48.2% in 2021 and 60.9% in 2022, whereas the proportion of adolescents aged 10–18 years decreased from 20.3% to 13.7% and 4.7%, respectively. The proportion of children aged 1–5 years also decreased from 37.3% in 2020 to 32.1% in 2021 and 30.0% in 2022, while children aged 6–9 years accounted for 8.5%, 6.0%, and 4.3% of admissions, respectively. Similar trends have been described in multinational pediatric cohorts, particularly during the Omicron wave, and are likely explained by lower admission thresholds for young infants, increasing immunity among older children, and evolving hospitalization policies rather than greater disease severity [35,36]. A similar demographic shift was reported by Bahl et al. in a multicenter American cohort, where the proportion of infants among hospitalized children increased from 16.7% during the Alpha-predominant period to 28.5% during the Omicron-predominant period. They also reported that adolescent admissions decreased from 39.1% to 22.1% [25]. When Zhu et al. analyzed 31,785 hospitalized children from 9 countries, they reported a decrease in median age from 5 years during earlier pandemic periods to 3 years during Omicron predominance [24]. Doenhardt et al. reported that 41.9% of 7375 hospitalized children in a predominantly German surveillance cohort were younger than one year [34]. These findings were also described in another cohort from Romania that included 613 infants hospitalized during the Omicron wave [37].

In contrast, sex distribution remained stable throughout the pandemic, consistent with previous reports showing only a modest male predominance among hospitalized children without a clear association with disease severity [38]. This finding is comparable to the results of Doenhardt et al., who reported 53.7% male patients, and Zhu et al., who reported a 52.3% male proportion [24,34]. We also observed a predominance of children from urban areas, particularly during the later pandemic years, probably reflecting referral patterns, healthcare accessibility, and higher testing availability rather than differences in susceptibility to SARS-CoV-2 infection. Previous Romanian research has documented changes in pediatric healthcare utilization during the pandemic, emphasizing the potential influence of healthcare system factors on hospitalization patterns [39].

Respiratory manifestations remained the predominant clinical presentation throughout the study period, although their pattern evolved over time, rising from 61.9% in 2020 to 70.2% in 2021 and 77.3% in 2022. We observed a progressive transition from predominantly isolated lower respiratory tract involvement during the early pandemic to more frequent combined upper and lower respiratory tract disease in 2021 and 2022, accompanied by improved oxygenation. Similar findings have been reported following the emergence of the Omicron variant, which was associated with greater transmissibility but lower pulmonary severity in children [40,41]. Comparable changes in pediatric respiratory presentations were described by Han et al. in a South Korean cohort of 1163 children. Their study found that pneumonia and prolonged fever were more frequent during the Delta wave, whereas the Omicron wave was characterized by younger patient age, higher fever, febrile seizures, and croup [33]. Sharma et al. and Brewster et al. also documented SARS-CoV-2-associated croup during Omicron predominance, supporting the observation that upper respiratory tract manifestations became increasingly recognized during this period [40,41].

Beyond respiratory involvement, gastrointestinal manifestations remained consistently common throughout the study period, affecting 75.4%, 74.4%, and 78.1% of patients in 2020, 2021, and 2022, respectively, without a statistically significant temporal difference. This finding supports the concept that pediatric COVID-19 is a multisystem disease rather than an isolated respiratory infection [8]. The proportions in our study are considerably higher than those reported in several pediatric studies. Akobeng et al., in a systematic review and meta-analysis, estimated a pooled prevalence of gastrointestinal symptoms of approximately 22.8%, with diarrhea, vomiting, and abdominal pain reported in 12.4%, 10.3%, and 5.4% of children, respectively [42]. The substantial difference between our findings and these pooled estimates may be related to variations in the definition and recording of gastrointestinal manifestations, the characteristics of hospitalized populations, and the inclusion of broader symptom categories in our study. Gastrointestinal manifestations of SARS-CoV-2 infection may result from several mechanisms, including direct infection of intestinal epithelial cells, local inflammatory responses, and alterations in the composition and function of the gut microbiota [8,42,43,44,45,46,47]. Some studies have demonstrated that SARS-CoV-2 can infect and replicate in human intestinal enterocytes, with ACE2-mediated viral entry facilitated by host proteases such as TMPRSS2 and TMPRSS4 and they also provided evidence of intestinal infection and alterations in local inflammatory responses [45,46,47]. Beyond these mechanisms, some experimental investigations have explored the possibility of bacteriophage-like interactions between SARS-CoV-2 and intestinal bacteria. Brogna et al. initially reported observations suggesting potential interactions between SARS-CoV-2 and bacterial components of the human gut microbiota, including the detection of viral material associated with bacterial cells [48]. Petrillo et al. provided additional experimental observations, including electron microscopy findings and molecular analyses, which the authors interpreted as supporting a possible bacteriophage-like behavior of SARS-CoV-2 [44]. Brogna et al. investigated the association between early antibiotic treatment and clinical recovery in a retrospective cohort of 211 patients with COVID-19, reporting shorter recovery times among patients who received antibiotics early in the course of infection [49].

Neuromuscular manifestations decreased substantially in our cohort, from 43.2% in 2020 to 21.4% in 2021 and 12.0% in 2022. This finding suggests a temporal change in the distribution of neurological and systemic symptoms. Han et al. reported a greater frequency of febrile seizures during the Omicron wave, illustrating that individual neurological manifestations may follow different temporal patterns [33]. Differences in symptom definitions, age distributions, and the relative contribution of nonspecific symptoms such as headache or myalgia may explain discrepancies between studies. Clinically significant cardiovascular involvement was uncommon, with tachycardia representing the most frequent finding and likely reflecting systemic illness rather than primary myocardial injury. This should be distinguished from severe cardiac complications and multisystem inflammatory syndrome in children, which have been characterized in other pediatric cohorts [17]. Overall, our findings indicate that although the clinical phenotype of pediatric COVID-19 evolved during successive pandemic waves, the disease remained predominantly mild to moderate in hospitalized children.

Temporal changes were also observed in laboratory parameters and the prevalence of underlying comorbidities, reflecting the evolving characteristics of the hospitalized pediatric population throughout the pandemic. While hematological indices varied across the study years, inflammatory markers remained relatively stable, emphasizing the importance of interpreting laboratory findings in conjunction with the overall clinical presentation rather than in isolation. Median leukocyte counts decreased from 10,988.5 cells/µL in 2020 to 7395 cells/µL in 2021 and 7000 cells/µL in 2022, while median hemoglobin values decreased from 11.8 to 10.9 g/dL. Han et al. similarly reported differences in hematological abnormalities across pandemic waves, including age-dependent changes in leukopenia, neutropenia, and lymphopenia [33]. Our study did not establish whether the observed laboratory differences were independently associated with viral variants, patient age, or disease severity. The relatively stable inflammatory markers in our cohort further suggest that changes in individual hematological indices should be interpreted within the broader clinical context.

The decreasing proportion of children with chronic comorbidities admitted during the later pandemic years further suggests that broader community transmission and evolving hospitalization practices increasingly resulted in the admission of otherwise healthy children. The proportion of children without underlying comorbidities increased from 66.9% in 2020 to 82.8% in 2022. Overall, 72.3% of our patients had no documented pre-existing medical conditions. This proportion is similar to that reported by Doenhardt et al., in which their nationwide surveillance study identified underlying comorbidities in almost 27% of hospitalized children [34]. Neurological disorders were the most frequent comorbidity category in our cohort (7.5%), followed by gastrointestinal diseases (6.4%) and cardiovascular disorders (4.0%). Although the prevalence of several comorbidity categories decreased during the later study years, these differences were not consistently statistically significant.

Our study has several strengths, including the relatively large cohort, the longitudinal assessment of three consecutive pandemic years, and the comprehensive evaluation of epidemiological, clinical, and laboratory characteristics within a single tertiary pediatric referral center. However, the retrospective single-center design, the absence of routine viral sequencing, and changes in testing and admission policies should be considered when interpreting our findings. Age and hospitalization duration were available only as categorical variables, preventing the calculation of exact means and standard deviations. Detailed information on treatment regimens, medication duration, and time to clinical recovery was not systematically collected, limiting our ability to assess therapeutic practices and recovery outcomes across the study years. Despite these limitations, our study provides valuable longitudinal evidence on the evolving characteristics of pediatric SARS-CoV-2 infection and may contribute to improved risk stratification, clinical decision-making, and preparedness for future respiratory viral outbreaks.

## 5. Conclusions

In conclusion, the epidemiological, clinical, and laboratory characteristics in hospitalized children with SARS-CoV-2 infection in our cohort evolved substantially between 2020 and 2022, although the overall disease course remained predominantly mild to moderate. The proportion of infants increased from 33.9% in 2020 to 60.9% in 2022, while adolescent admissions declined from 20.3% to 4.7%. Respiratory manifestations became more frequent (61.9% vs. 77.3%), whereas neuromuscular manifestations decreased substantially (43.2% vs. 12.0%). The persistence of gastrointestinal manifestations throughout the study period also emphasizes the importance of understanding the mechanisms underlying extrapulmonary involvement. In this context, emerging experimental evidence suggests potential bacteriophage-like interactions between SARS-CoV-2 and the intestinal microbiota. The proportion of children without underlying comorbidities increased from 66.9% in 2020 to 82.8% in 2022, suggesting a changing profile of hospitalized patients.

Our findings provide longitudinal evidence from a tertiary pediatric referral center in Eastern Europe, highlighting the dynamic nature of pediatric COVID-19 across successive pandemic years.

Continued epidemiological surveillance and age-specific risk stratification remain essential to optimize the management of hospitalized children and to improve preparedness for future emerging respiratory viral infections.

## Figures and Tables

**Table 1:** Demographic and epidemiological characteristics of the patients included in the study.

Characteristic	2020 (*n* = 118)	2021 (*n* = 168)	2022 (*n* = 233)	Total (*n* = 519)	*p*-Value
**Age group,** ***n* (%)**					<0.001
<1 year	40 (33.9%)	81 (48.2%)	142 (60.9%)	263 (50.7%)	
1–5 years	44 (37.3%)	54 (32.1%)	70 (30.0%)	168 (32.4%)	
6–9 years	10 (8.5%)	10 (6.0%)	10 (4.3%)	30 (5.8%)	
10–18 years	24 (20.3%)	23 (13.7%)	11 (4.7%)	58 (11.2%)	
**Sex,** ***n* (%)**					0.681
Female	45 (38.1%)	72 (42.9%)	99 (42.5%)	216 (41.6%)	
Male	73 (61.9%)	96 (57.1%)	134 (57.5%)	303 (58.4%)	
**Residence,** ***n* (%)**					0.020
Urban	79 (66.9%)	98 (58.3%)	167 (71.7%)	344 (66.3%)	
Rural	39 (33.1%)	70 (41.7%)	66 (28.3%)	175 (33.7%)	
**Hospitalization duration,** ***n* (%)**					<0.001
1–3 days	105 (89.0%)	42 (25.0%)	63 (27.0%)	210 (40.5%)	
4–7 days	13 (11.0%)	58 (34.5%)	139 (59.7%)	210 (40.5%)	
>7 days	0 (0.0%)	68 (40.5%)	31 (13.3%)	99 (19.1%)	
**Epidemiological contact,** ***n* (%)**					0.14
No known contact	58 (49.2%)	78 (46.4%)	118 (50.6%)	254 (48.9%)	
Household contact	60 (50.8%)	85 (50.6%)	113 (48.5%)	258 (49.7%)	
School contact	0 (0.0%)	5 (3.0%)	1 (0.4%)	6 (1.2%)	
Other	0 (0.0%)	0 (0.0%)	1 (0.4%)	1 (0.2%)	

**Table 2:** Comparison of demographic and hospitalization characteristics by sex.

Characteristic	Female (*n* = 216)	Male (*n* = 303)	Total (*n* = 519)	*p*-Value
Pandemic year, n (%)				0.681
2020	45 (20.8%)	73 (24.1%)	118 (22.7%)	
2021	72 (33.3%)	96 (31.7%)	168 (32.4%)	
2022	99 (45.8%)	134 (44.2%)	233 (44.9%)	
Age group, n (%)				0.503
<1 year	108 (50.0%)	155 (51.2%)	263 (50.7%)	
1–5 years	73 (33.8%)	95 (31.4%)	168 (32.4%)	
6–9 years	15 (6.9%)	15 (5.0%)	30 (5.8%)	
10–18 years	20 (9.3%)	38 (12.5%)	58 (11.2%)	
Residence, n (%)				0.414
Urban	148 (68.5%)	196 (64.7%)	344 (66.3%)	
Rural	68 (31.5%)	107 (35.3%)	175 (33.7%)	
Hospitalization duration, n (%)				0.170
1–3 days	78 (36.1%)	132 (43.6%)	210 (40.5%)	
4–7 days	97 (44.9%)	113 (37.3%)	210 (40.5%)	
>7 days	41 (19.0%)	58 (19.1%)	99 (19.1%)	

**Table 3:** Comparative clinical characteristics of pediatric COVID-19 cases (2020–2022).

Variable (%/SE)	2020 (*n* = 118)	2021 (*n* = 168)	2022 (*n* = 233)	Total (*n* = 519)	*p*-Value
**Fever (%)**	75.4 (4.0)	81.0 (3.0)	89.3 (2.0)	83.4 (1.6)	0.003
**Fever duration (%)**					0.003
**No fever**	24.5 (4.0)	19.1 (3.0)	12.0 (2.1)	17.2 (1.7)	
**1–3 days**	65.3 (4.4)	60.7 (3.8)	75.1 (2.8)	68.2 (2.0)	
**>3 days**	10.2 (2.8)	20.2 (3.1)	12.9 (2.2)	14.6 (1.5)	
**General condition at admission (%)**					
**Good**	7.6 (2.4)	0.6 (0.6)	1.7 (0.8)	2.7 (0.7)	<0.001
**Satisfactory**	89.0 (2.9)	58.9 (3.8)	55.4 (3.3)	64.2 (2.1)	
**Moderate**	3.4 (1.7)	39.9 (3.8)	42.9 (3.2)	32.9 (2.1)	
**Severe**	0.0 (0.0)	0.6 (0.6)	0.0 (0.0)	0.2 (0.2)	
**SpO_2_ (%)**					<0.001
**>95%**	78.0 (3.8)	86.3 (2.7)	94.4 (1.5)	88.1 (1.4)	
**90–94%**	20.3 (3.7)	13.1 (2.6)	5.6 (1.5)	11.4 (1.4)	
**<90%**	1.7 (1.2)	0.6 (0.6)	0.0 (0.0)	0.6 (0.3)	
**Respiratory manifestations (%)**	61.9 (4.5)	70.2 (3.5)	77.3 (2.7)	71.5 (2.0)	0.01
**Digestive manifestations (%)**	75.4 (4.0)	74.4 (3.4)	78.1 (2.7)	75.1 (1.9)	0.66
**Cardiovascular manifestations (%)**	37.3 (4.5)	25.0 (3.3)	27.5 (2.9)	28.9 (2.0)	0.06
**Neuromuscular manifestations (%)**	43.2 (4.6)	21.4 (3.2)	12.0 (2.1)	22.2 (1.8)	<0.001
**Reno-urinary manifestations (%)**	2.5 (1.4)	3.6 (1.4)	6.9 (1.7)	4.8 (0.9)	0.12
**Cutaneous eruptions (%)**	5.1 (2.0)	11.3 (2.4)	6.9 (1.7)	7.9 (1.2)	0.12

**Table 4:** Comparative paraclinical findings across study years.

Variable	2020 (*n* = 118)	2021 (*n* = 168)	2022 (*n* = 233)	Total (*n* = 519)	*p*-Value
**Leukocytes, median (CI)**	10,988.5 (10,072–11,785.7)	7395 (7669.8–8962.8)	7000 (7432.3–8539.8)	7880 (8372.4–9152.0)	<0.001
**Neutrophils, median (CI)**	2984.5 (3170.3–4259.3)	3015 (3224.2–4188.9)	2630 (3045.8–3834.9)	2750 (3323.8–3853.9)	0.45
**Lymphocytes, median (CI)**	3496.5 (3537.0–4399.9)	3210 (3488.4–4518.9)	2770 (3187.7–3946.2)	3180 (3542.8–4056.3)	0.03
**Hemoglobin, median (CI)**	11.8 (11.4–11.9)	11.2 (9.5–16.4)	10.9 (10.6–11.7)	11.2 (10.7–13.0)	0.02
**C-reactive protein, median (CI)**	13.5 (9.4–17.6)	14.4 (10.6–18.2)	11.5 (8.5–14.5)	12.9 (10.9–14.9)	0.08

**Table 5:** Distribution of comorbidities across the 3 study years.

Comorbidity	2020 (*n* = 118)	2021 (*n* = 168)	2022 (*n* = 233)	Total (*n* = 519)	*p*-Value
No comorbidities	79 (66.9%)	103 (61.3%)	193 (82.8%)	375 (72.3%)	<0.001
Cardiovascular	2 (1.7%)	11 (6.5%)	8 (3.4%)	21 (4.0%)	0.100
Respiratory	5 (4.2%)	7 (4.2%)	5 (2.1%)	17 (3.3%)	0.427
Gastrointestinal	9 (7.6%)	16 (9.5%)	8 (3.4%)	33 (6.4%)	0.039
Neurological	14 (11.9%)	12 (7.1%)	13 (5.6%)	39 (7.5%)	0.105
Immunological/hematological	3 (2.5%)	10 (6.0%)	4 (1.7%)	17 (3.3%)	0.055
Diabetes/obesity	1 (0.8%)	4 (2.4%)	0 (0.0%)	5 (1.0%)	0.054
Reno-urinary	2 (1.7%)	0 (0.0%)	5 (2.1%)	7 (1.3%)	0.172
Psychiatric	3 (2.5%)	4 (2.4%)	2 (0.9%)	9 (1.7%)	0.384
Ophthalmological	0 (0.0%)	3 (1.8%)	0 (0.0%)	3 (0.6%)	0.043
Genetic syndromes	0 (0.0%)	0 (0.0%)	2 (0.9%)	2 (0.4%)	0.292

## Data Availability

The datasets generated and analyzed during the current study are available from the corresponding author upon reasonable request, subject to institutional regulations governing patient confidentiality.
